# Prevalence, Regional Variations, and Predictors of Overweight, Obesity, and Hypertension Among Healthy Reproductive-Age Indian Women: Nationwide Cross-Sectional Polycystic Ovary Syndrome Task Force Study

**DOI:** 10.2196/43199

**Published:** 2023-09-06

**Authors:** Mohd Ashraf Ganie, Subhankar Chowdhury, Vanita Suri, Beena Joshi, Prasanta Kumar Bhattacharya, Sarita Agrawal, Neena Malhotra, Rakesh Sahay, Puthiyaveettil Khadar Jabbar, Roya Rozati, Imtiyaz Ahmad Wani, Amlin Shukla, Taruna Arora, Haroon Rashid

**Affiliations:** 1 Department of Endocrinology, Sher-i-Kashmir Institute of Medical Sciences Srinagar India; 2 Department of Endocrinology, Institute of Postgraduate Medical Education & Research Kolkata India; 3 Department of Obstetrics & Gynaecology, Postgraduate Institute of Medical Education and Research Chandigarh India; 4 Department of Operational Research, National Institute for Research in Reproductive Health, Indian Council of Medical Research (ICMR) Mumbai India; 5 Department of General Medicine, North Eastern Indira Gandhi Regional Institute of Health and Medical Sciences Meghalaya India; 6 Department of Obstetrics & Gynaecology, All India Institute of Medical Sciences Chatisgarh India; 7 Department of Obstetrics & Gynaecology, All India Institute of Medical Sciences New Delhi India; 8 Department of Endocrinology, Osmania Medical College Hyderabad India; 9 Department of Endocrinology, Government Medical College Kerala India; 10 Department of Obstetrics & Gynaecology, Maternal Health & Research Trust Hyderabad India; 11 Reproductive Biology and Maternal Health, Indian Council of Medical Research (ICMR) New Delhi India

**Keywords:** Indian Council of Medical Research–polycystic ovary syndrome study, ICMR-PCOS study, noncommunicable diseases, disease surveillance, obesity, hypertension, anthropometry, healthy reproductive-age women, socioeconomic status

## Abstract

**Background:**

A clear understanding of the anthropometric and sociodemographic risk factors related to BMI and hypertension categories is essential for more effective disease prevention, particularly in India. There is a paucity of nationally representative data on the dynamics of these risk factors, which have not been assessed among healthy reproductive-age Indian women.

**Objective:**

This cross-sectional polycystic ovary syndrome (PCOS) task force study aimed to assess the anthropometric and sociodemographic characteristics of healthy reproductive-age Indian women and explore the association of these characteristics with various noncommunicable diseases.

**Methods:**

We conducted a nationwide cross-sectional survey from 2018 to 2022 as part of the Indian Council of Medical Research–PCOS National Task Force study, with the primary aim of estimating the national prevalence of PCOS and regional phenotypic variations among women with PCOS. A multistage random sampling technique was adopted, and 7107 healthy women (aged 18-40 years) from 6 representative geographical zones of India were included in the study. The anthropometric indices and sociodemographic characteristics of these women were analyzed. Statistical analysis was performed to assess the association between exposure and outcome variables.

**Results:**

Of the 7107 study participants, 3585 (50.44%) were from rural areas and 3522 (49.56%) were from urban areas. The prevalence of obesity increased from 8.1% using World Health Organization criteria to 40% using the revised consensus guidelines for Asian Indian populations. Women from urban areas showed higher proportions of overweight (524/1908, 27.46%), obesity (775/1908, 40.62%), and prehypertension (1008/1908, 52.83%) categories. A rising trend of obesity was observed with an increase in age. Women aged 18 to 23 years were healthy (314/724, 43.4%) and overweight (140/724, 19.3%) compared with women aged 36 to 40 years with obesity (448/911, 49.2%) and overweight (216/911, 23.7%). The proportion of obesity was high among South Indian women, with 49.53% (531/1072) and 66.14% (709/1072), using both World Health Organization criteria and the revised Indian guidelines for BMI, respectively. BMI with waist circumference and waist-to-height ratio had a statistically significant linear relationship (*r*=0.417; *P*<.001 and *r*=0.422; *P*<.001, respectively). However, the magnitude, or strength, of the association was relatively weak (0.3<|*r*|<0.5). Statistical analysis showed that the strongest predictors of being overweight or obese were older age, level of education, wealth quintile, and area of residence.

**Conclusions:**

Anthropometric and sociodemographic characteristics are useful predictors of overweight- and obesity-related syndromes, including prehypertension, among healthy Indian women. Increased attention to the health of Indian women from public health experts and policy makers is warranted. The findings of this study can be leveraged to offer valuable insights, informing health decision-making and targeted interventions that mitigate risk factors of overweight, obesity, and hypertension.

**International Registered Report Identifier (IRRID):**

RR2-10.2196/23437

## Introduction

### Background

Over the past few decades, a disturbing epidemiological transition has revealed that chronic noncommunicable diseases (NCDs) are on the rise both at the global and regional levels. The etiology of NCDs has remained vague and has been attributed to many factors, ranging from genetic predispositions to human lifestyles. The lack of physical exercise, high BMI, increased blood pressure (BP), and obesity are a few factors that affect people’s health and predispose them to the risk of NCDs [1,2]. Obesity has now emerged as a major worldwide health concern and is probably the main cause of the deranged metabolism leading to metabolic syndrome, including insulin resistance (IR), type 2 diabetes mellitus, hypertension, and dyslipidemia [3,4]. Scientific evidence reveals that women with obesity are more vulnerable to infertility, miscarriage, and other reproductive complications [5]. Researchers have brought to light the fact that a fast-paced lifestyle has taken a toll on human health and is a contributor to the rise of various diseases and disorders. The issue of obesity and overweight has increased to epidemic proportions; one-third of the global population is overweight or obese, and according to the global burden of disease, >4 million people die each year as a result of these issues. According to the Indian Council of Medical Research (ICMR)–India Diabetes study 2015, the prevalence rates of obesity and central obesity vary from 11.8% to 31.3% and from 16.9% to 36.3%, respectively [6]. Another cross-sectional study reported an age-adjusted prevalence of hypertension of 10.9% in women, 12.5% in urban areas, and 10.6% in rural areas [7]. Recent reports from the World Health Organization (WHO) revealed that, among adults aged ≥18 years, 13% are obese and 39% are overweight [8]. The 2019 to 2021 National Family Health Survey (NFHS)–5 anthropometric data revealed that 19% of women aged between 15 and 49 years are underweight, 24% are overweight or obese, and 57% have a BMI within the normal range [9].

A global strategy for improving health, early detection, and prevention of disease epidemics can be developed by adopting appropriate, accessible, affordable, and scalable health solutions. Anthropometry is the single most universally applicable, inexpensive, noninvasive, and reliable method available for the assessment of body composition, size, and proportions among other methods such as biochemical and radiological assessments. Anthropometric indices are being used to predict the increased risk of several diseases at both the individual and population levels [10-12]. These indices are combinations of anthropometric measurements (BMI, waist circumference [WC], and waist-to-hip ratio [WHR]) that are vital for the interpretation of measurements and assessment of NCD risk factors in the population [13,14]. The most frequently used anthropometric indices are BMI and WC as predictors of overweight- and obesity-related syndromes, including hypertension. The existing literature includes waist-to-height ratio (WHtR) as an additional anthropometric indicator for the assessment of overweight- and obesity-related syndromes, including hypertension [9,15,16]. Several epidemiological studies have consistently reported an association between hypertension and BMI, WC, WHR, and WHtR [17,18].

The correlation between obesity and hypertension is a major concern worldwide. The prevalence of obesity in India is not uniform and varies according to age, gender, geographical environment, and socioeconomic status (SES). Furthermore, many sociodemographic indicators such as education, SES, and occupation and ethnic and racial differences are linked to health status through multiple pathways; however, their clear relationships have not yet been fully elucidated and hitherto remain obscure. The NFHS conducted in India has depicted substantial differences using WHO criteria in the prevalence of overweight and obesity among adults across regions, implying the role of geographic and regional factors. However, there is a paucity of similar literature among reproductive age–matched healthy women using revised consensus guidelines for Indian populations. Myriad previous studies have revealed that the prevalence of prehypertension is an important risk and predisposing factor for developing hypertension in the future [19]. Making use of Western anthropometric standards for low- and middle-income countries (LMICs) has remained controversial [20]; hence, country-specific anthropometric data become indispensable in this regard. It has been very well acknowledged that no standard is universally acceptable as the definition of indices such as optimal growth differs with ethnicity and genetic makeup. In addition, these anthropometric parameters are not uniform and vary considerably from population to population. However, these anthropometric indicators have high predictive value for assessing risk factors of NCDs, thus offering a robust mechanism for the identification of risk factors in relatively easy and cost-effective ways in a local setting.

### Objectives

The pattern of overall lifestyle varies from region to region, which necessitates a comprehensive national-level research study, especially among young, healthy reproductive-age women across diverse regions of the country. India has called for the highest priority for the health needs of women, who constitute nearly 50% of the country’s population. Taking a cue, the ICMR has taken an initiative under the ICMR–polycystic ovary syndrome (PCOS) Task Force study to address women-centric issues, wherein attention has also been paid to the assessment of various aberrations among women from representative regions of India using anthropometric indices. As the prevalence of overweight, obesity, and hypertension is rising in LMICs such as India, it becomes imperative to understand the dynamics of the various sociodemographic factors and their association with the incidence of overweight, obesity, and BP profile among age-matched healthy women from representative regions of India. With this endeavor, this study attempted to address the gaps in NFHS anthropometric data using nationally representative data through the adoption of revised country-specific guidelines.

## Methods

### Setting

We used data from the ICMR-PCOS Task Force study conducted across representative regions of India [21]. The data were collected from both rural and urban areas in 6 geographical zones of the country.

### Ethics Approval

The study was conducted in accordance with the Helsinki Declaration of 1975 and has been approved by the institutional ethics committees at all 10 study sites as: 131/IEC-SKIMS/2017-101- Sher-i-Kashmir Institute of Medical Sciences, Srinagar; PGI/IEC/2017/47-Postgraduate Institute of Medical Education and Research, Chandigarh;IEC-34/09.02.2017- All India Institute of Medical Sciences, Delhi; IEC/2017/057- Institute of Post Graduate Medical Education and Research, Kolkata; NEIGR/IEC/2018/02- North Eastern Indira Gandhi Regional Institute of Health and Medical Sciences; ECR/300/Inst/AP/2017-Osmania Medical College, Hyderabad; IEC/MHRT/302- Maternal Health and Research Trust, Hyderabad;173/IEC-AIIMSRPR/2017- All India Institute of Medical Sciences, Raipur; D/ICEC/Sci-33/37/2017- National Institute for Research in Reproductive Health, Mumbai and IEC.No.07/09/2017/MCT- Government Medical College, Trivandrum.

### Informed Consent and Safety Measures

Written informed consent was obtained from the participants, ensuring their understanding of the purpose of the study and the obligations and possible consequences of their participation. During the data collection process, face masks, hand gloves, and hand sanitizer were provided to the data collection team and study participants to protect the data collectors and participants from COVID-19 infection.

### Study Sites

This study involved the participation of the following sites: All India Institute of Medical Sciences, Delhi; All India Institute of Medical Sciences, Raipur; Government Medical College, Trivandrum; Institute of Post Graduate Medical Education and Research, Kolkata; Maternal Health and Research Trust, Hyderabad; North Eastern Indira Gandhi Regional Institute of Health and Medical Sciences, Shillong; National Institute for Research in Reproductive Health, Mumbai; Osmania Medical College, Hyderabad; Postgraduate Institute of Medical Education and Research, Chandigarh; and Sher-i-Kashmir Institute of Medical Sciences, Srinagar.

### Sampling Design

This multicenter study involved the recruitment of apparently healthy women aged 18 to 40 years using a multistage sampling technique from randomly selected polling booths across urban and rural areas from the 6 zones (ie, North, South, East, West, Northeast, and Central India) of the country. The states and union territories were grouped into 6 geographical regions based on their sociocultural similarities, with a small change from the grouping (Table 1 shows the composition of each zone). Details of the sample size and sampling plan are available in our published protocol in *JMIR Research Protocols* [21].

**Table 1:** States and union territories in each region.

Region or zone	State and UT^a^
East	West Bengal
West	Maharashtra
North	Jammu and Kashmir (UT), Chandigarh (UT), and Delhi (UT)
Northeast	Meghalaya
Central	Chhattisgarh
South	Telangana

^a^UT: Union Territory.

### Study Population

**Figure 1:**
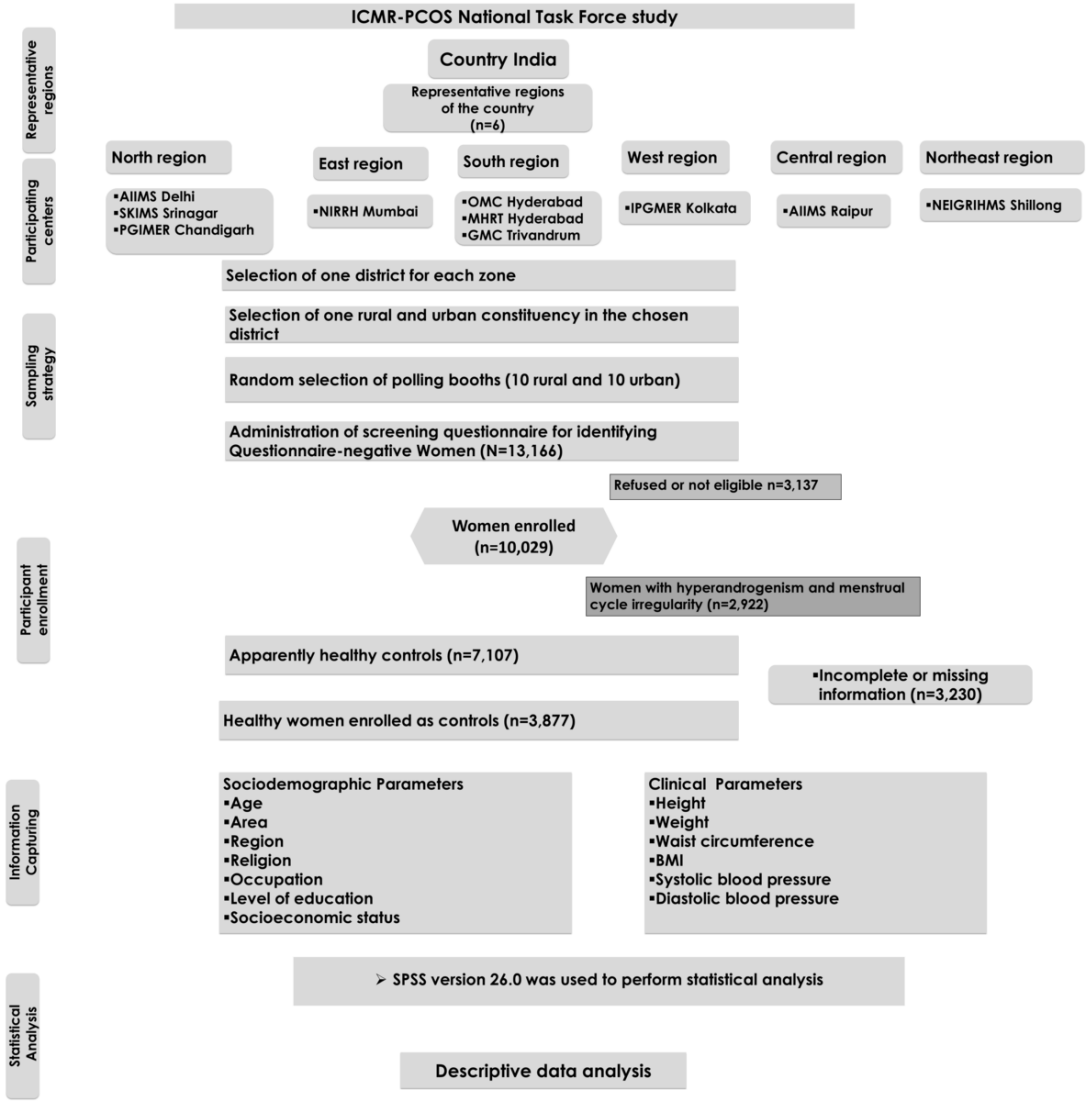
CONSORT (Consolidated Standards of Reporting Trials) diagram showing selection of participants in the cross-sectional polycystic ovary syndrome (PCOS) Task Force study. AIIMS: All India Institute of Medical Sciences; GMC: Government Medical College; ICMR: Indian Council of Medical Research; IPGMER: Institute of Post Graduate Medical Education and Research; MHRT: Maternal Health and Research Trust; NEIGRIHMS: North Eastern Indira Gandhi Regional Institute of Health and Medical Sciences; NIRRH: National Institute for Research in Reproductive Health; OMC: Osmania Medical College; PGIMER: Postgraduate Institute of Medical Education and Research; SKIMS: Sher-i-Kashmir Institute of Medical Sciences.

We approached a total of 13,166 women as per the voter ID list, of whom 3137 (23.83%) were either not eligible or refused to give consent and were excluded. Of the total 10,029 women, 2922 (29.14%) were excluded using the inclusion and exclusion criteria (Textbox 1). A total of 7107 women were enrolled as apparently healthy controls with complete sociodemographic details, and after considering the missing clinical information, our analysis included 3877 (54.55%) apparently healthy women from representative regions of the country (Figure 1).

**Inclusion and exclusion criteria.**

**Inclusion criteria**

- Healthy women aged between 18-40 years
- Women who were permanent residents of that area (>1 y)
- Willingness to participate in the study and sign an informed consent form

**Exclusion criteria**

- Participants with hypothyroidism, subclinical hypothyroidism, hyperthyroidism, hyperprolactinemia, type 2 diabetes, exogenous Cushing syndrome, premature ovarian failure, and hypopituitarism
- Pregnant or lactating women and those with cognitive or physical limitations that prevented them from answering the questionnaire

### Data Collection

Well-trained research staff collected the requisite data as appropriate at each study site. After obtaining written consent, an in-depth questionnaire was presented to each woman enrolled from the community (rural and urban) to obtain demographic information about her age, religion, marital status, level of education, SES, occupation, and type of family. Anthropometric data collected included height, weight, WHR, and WC. BP readings were taken at 5-minute intervals. The junior medical officers were responsible for the overall management of the field team at all sites. The investigators at all participating centers monitored the data collection process and checked the data during entry.

### Tools and Techniques

#### Sociodemographic Information

Sociodemographic information pertaining to age, sex, address, occupation, education, SES, marital status, and religion was obtained using a paper-based questionnaire. Information related to anthropometric and related parameters in study participants was obtained through in-person physical evaluation during community visits.

#### Anthropometric Measurements

Participants included in the study underwent a range of anthropometric measurements in accordance with the instruction manual designed before the initiation of the study. Trained research staff were engaged to take the measurements and record the clinical data from all participants, including age, body weight (kg), height (cm), BMI (kg/m^2^), WHR, and BP. The anthropometric measurements were performed using anthropometric tools (SECA 213, 813, and 203 scales) with similar specifications at all participating sites. The equipment was maintained and calibrated as per the manufacturer’s instructions. BMI was calculated using the following formula: *BMI = weight (kg) / height^2^ (m^2^)*. BP was measured 3 times with an automated sphygmomanometer in a sitting position after at least 5 minutes of rest.

Height was measured using a portable stadiometer (SECA 213) with precision nearest to 0.1 cm. Before measurement, the stadiometer was checked using calibration rods. During measurement, the participants were asked to take off their shoes. To ensure correct measurements, the shoulder, buttocks, calf, heel, and back of the head of each participant were to touch the vertical stand of the stadiometer.

Body weight was measured using a digital scale (SECA 813) with a precision of 0.1 kg. The accuracy of the scale was checked by weighing an object of known weight. The participants were asked to stand straight on the electronic weighing scale barefoot and with light clothing, and the weight displayed on the screen was recorded.

WC was measured using nonstretchable measuring tape (SECA), and the participants were asked to raise their clothing above the waist to avoid any interference with the measurements. WC was measured on bare skin with the participant in a standing position and at the narrowest indentation at the midpoint between the costal margin and iliac crest.

BP was measured using an automatic BP monitor (OMRON HEM-7124). To ensure adherence to WHO criteria for the measurement of BP, the participants were asked to sit on a chair and allowed to rest as required with their feet on the ground and their palm facing upward. All BP measurements were performed preferably on the left arm, ensuring that the cuff was wrapped over the bare upper arm, 2 to 3 cm above the antecubital fossa. The average of 3 consecutive measurements was taken as the BP of the participant.

### Categorization of Variables

The primary outcome variables of our study included prevalence of overweight, obesity, and hypertension. The variables used were age; place of residence; geographical region; marital status; occupation; SES; and biological risk factors such as BMI, WC, and WHtR of the study participants. BMI was calculated as the ratio of weight (in kg) to the square of height (in m; *BMI = weight / height^2^*). For the WHR, WC was divided by hip circumference. BMI was categorized as healthy, underweight, overweight, and obese. According to the WHO classification of obesity, BMI is categorized as healthy (<24.9 kg/m^2^), overweight (25-29.9 kg/m^2^), and obese (>30 kg/m^2^) [22]. However, in this study, BMI was calculated based on the revised consensus guidelines for India [23,24]. Using this scale, the participants were categorized as underweight (<18.5 kg/m^2^), healthy (18.5-22.9 kg/m^2^), overweight (23.0-24.9 kg/m^2^), and obese (≥25 kg/m^2^).

The explanatory variables were selected based on published reports and the data set’s structure. The study collected information pertaining to the participants’ age (18-23, 24-29, 30-35, and 36-40 y), place of residence (urban and rural), geographical location (East, West, North, South, Northeast, and Central India), marital status, educational level (ie, illiterate, up to primary school, up to secondary school, up to high school, and above), SES using the BG Prasad scale (eg, lower class, middle class, lower middle class, and upper class), and religion (ie, Hindu, Muslim, Christian, Sikh, and others).

### Definition and Categorization of Hypertension

Hypertension was defined according to the Eighth Joint National Committee on Prevention, Detection, Evaluation, and Treatment of High Blood Pressure (JNC 8) criteria. All the participants with a systolic BP (SBP) of ≥140 mm Hg or a diastolic BP (DBP) of ≥90 mm Hg or who reported taking medication at present for the treatment of high BP were classified as having hypertension. As per the JNC 8, (1) stage-1 hypertension was defined as an SBP of 140 to 159 mm Hg or DBP of 90 to 99 mm Hg, and (2) prehypertension was defined as an SBP of >120 mm Hg but less than the cutoff for hypertension or DBP between 80 and 89 mm Hg in the absence of a diagnosis of hypertension or treatment with medication for high BP [23].

### Statistical Analysis

The data from all study participants were entered into Excel (Microsoft Corp) sheets, and we used SPSS (version 26.0; IBM Corp) for data analysis. Descriptive statistical analysis was performed, and categorical variables were stratified into groups and then presented as numbers and percentages. Pearson correlation analysis was performed between BMI and WC and WHtR. A logistic regression model was used to determine the influence of demographic characteristics on general and central obesity. Variables included in the multivariate logistic regression analysis were age, region, SES, education, religion, marital status, and occupation. The data on the distribution of BMI categories using WHO criteria and the revised consensus guidelines for India were presented using bar charts. A 2-tailed *P* value of <.05 was considered statistically significant.

## Results

### Description

Apparently healthy women who met the inclusion criteria were enrolled as controls in the study. The sociodemographic information (age, sex, address, occupation, education, SES, marital status, and religion) of the study participants is shown in Table 2. Of the 7107 study participants, 3585 (50.44%) were from rural areas and 3522 (49.56%) were from urban areas. Most of the women (2323/7107, 32.69%) were aged 30 to 35 years, followed by 24 to 29 years (1869/7107, 26.3%), 36 to 40 years (1609/7107, 22.64%), and 18 to 23 years (1306/7107, 18.38%). Most women (4851/7107, 68.26%) were Hindu, followed by Muslim (1300/7107, 18.29%), Christian (837/7107, 11.78%), Sikh (88/7107, 1.24%), and other religions (31/7107, 0.44%). Approximately 73.8% (5245/7107) of women were married, and 24.22% (1721/7107) were unmarried, with approximately 1.98% (141/7107) of women being either widowed or un-remarried. Among the enrolled women, 22.37% (1590/7107) had a high school education, 18.62% (1323/7107) had a middle school education, 20.22% (1437/7107) had a postgraduate education, only 2.41% (171/7107) had received a professional education or honors, and 14.65% (1041/7107) were uneducated. Furthermore, 82.43% (5858/7107) of women were unemployed or unskilled, and 2.41% (171/7107) were in some profession. Most of them (2176/7107, 30.62%) belonged to the middle class, followed by the lower-middle class (1721/7107, 24.22%). Of all the enrolled women, 13.02% (925/7107) belonged to the upper class, 22.39% (1591/7107) belonged to the upper-middle class, and 9.62% (684/7107) belonged to the lower class.

**Table 2:** Sociodemographic characteristics of the study population from representative regions of the country (n=7107).

Variable		North (n=2216), n (%)	Northeast (n=829), n (%)	East (n=744), n (%)	Central (n=681), n (%)	South (n=1690), n (%)	West (n=947), n (%)	Total, n (%)
**Age group (years)**								
	18-23	441 (19.9)	218 (26.3)	120 (16.1)	218 (32)	252 (14.9)	57 (6)	1306 (18.4)
	24-29	496 (22.4)	267 (32.2)	199 (26.7)	203 (29.8)	481 (28.5)	223 (23.5)	1869 (26.3)
	30-35	765 (34.5)	221 (26.7)	260 (34.9)	198 (29.1)	530 (31.4)	349 (36.9)	2323 (32.7)
	36-40	514 (23.2)	123 (14.8)	165 (22.2)	62 (9.1)	427 (25.3)	318 (33.6)	1609 (22.6)
**Area of residence**								
	Rural	1152 (52)	431 (52)	391 (52.6)	294 (43.2)	822 (48.6)	495 (52.3)	3585 (50.4)
	Urban	1064 (48)	398 (48)	353 (47.4)	387 (56.8)	868 (51.4)	452 (47.7)	3522 (49.6)
**Religion**								
	Hindu	1314 (59.3)	123 (14.8)	481 (64.7)	638 (93.7)	1390 (82.2)	905 (95.6)	4851 (68.3)
	Muslim	817 (36.9)	9 (1.1)	263 (35.3)	25 (3.7)	164 (9.7)	22 (2.3)	1300 (18.3)
	Christian	3 (0.1)	679 (81.9)	0 (0)	12 (1.8)	136 (8)	7 (0.7)	837 (11.8)
	Sikh	82 (3.7)	0 (0)	0 (0)	4 (0.6)	0 (0)	2 (0.2)	88 (1.2)
	Other	0 (0)	18 (2.2)	0 (0)	2 (0.3)	0 (0)	11 (1.2)	31 (0.4)
**Marital status**								
	Unmarried	609 (27.5)	317 (38.2)	113 (15.2)	226 (33.2)	236 (14)	220 (23.2)	1721 (24.2)
	Married	1585 (71.5)	473 (57.1)	630 (84.7)	447 (65.6)	1406 (83.2)	704 (74.3)	5245 (73.8)
	Un-remarried	12 (0.5)	27 (3.3)	0 (0)	2 (0.3)	12 (0.7)	13 (1.4)	66 (0.9)
	Widowed	10 (0.5)	12 (1.4)	1 (0.1)	6 (0.9)	36 (2.1)	10 (1.1)	75 (1.1)
**Level of education**								
	Illiterate	348 (15.7)	25 (3)	274 (36.8)	28 (4.1)	221 (13.1)	145 (15.3)	1041 (14.6)
	Primary school certificate	188 (8.5)	97 (11.7)	192 (25.8)	50 (7.3)	106 (6.3)	78 (8.2)	711 (10)
	Middle school certificate	368 (16.6)	187 (22.6)	243 (32.7)	137 (20.1)	233 (13.8)	155 (16.4)	1323 (18.6)
	High school certificate	546 (24.6)	208 (25.1)	24 (3.2)	158 (23.2)	481 (28.5)	173 (18.3)	1590 (22.4)
	Intermediate or post–high school diploma	221 (10)	132 (15.9)	8 (1.1)	127 (18.6)	231 (13.7)	112 (11.8)	831 (11.7)
	Graduate or postgraduate	531 (24)	174 (21)	3 (0.4)	178 (26.1)	296 (17.5)	255 (26.9)	1437 (20.2)
	Profession or honors	14 (0.6)	3 (0.4)	0 (0)	3 (0.4)	122 (7.2)	29 (3.1)	171 (2.4)
**Occupation**								
	Unemployed	1851 (83.5)	517 (62.4)	133 (17.9)	537 (78.9)	1177 (69.6)	563 (59.5)	4778 (67.2)
	Unskilled worker	71 (3.2)	211 (25.5)	388 (52.2)	67 (9.8)	208 (12.3)	135 (14.3)	1080 (15.2)
	Semiskilled worker	74 (3.3)	43 (5.2)	212 (28.5)	51 (7.5)	110 (6.5)	81 (8.6)	571 (8)
	Skilled worker	104 (4.7)	15 (1.8)	11 (1.5)	21 (3.1)	86 (5.1)	72 (7.6)	309 (4.3)
	Arithmetic skill job	8 (0.4)	0 (0)	0 (0)	2 (0.3)	27 (1.6)	25 (2.6)	62 (0.9)
	Semiprofessional	24 (1.1)	13 (1.6)	0 (0)	3 (0.4)	42 (2.5)	27 (2.9)	109 (1.5)
	Professional	64 (2.9)	23 (2.8)	0 (0)	0 (0)	40 (2.4)	44 (4.6)	171 (2.4)
	Other	20 (0.9)	7 (0.8)	0 (0)	0 (0)	0 (0)	0 (0)	27 (0.4)
**Socioeconomic status**								
	Upper class	180 (8.1)	5 (0.6)	17 (2.3)	163 (23.9)	333 (19.7)	227 (24)	925 (13)
	Upper-middle class	431 (19.4)	15 (1.8)	466 (62.6)	204 (30)	265 (15.7)	210 (22.2)	1591 (22.4)
	Middle class	541 (24.4)	320 (38.6)	234 (31.5)	180 (26.4)	714 (42.2)	187 (19.7)	2176 (30.6)
	Lower-middle class	601 (27.1)	488 (58.9)	27 (3.6)	105 (15.4)	289 (17.1)	211 (22.3)	1721 (24.2)
	Lower class	463 (20.9)	1 (0.1)	0 (0)	29 (4.3)	89 (5.3)	112 (11.8)	694 (9.8)
**Type of family**								
	Living alone	70 (3.2)	3 (0.4)	0 (0)	10 (1.5)	57 (3.4)	7 (0.7)	147 (2.1)
	Nuclear	1346 (60.7)	723 (87.2)	569 (76.5)	380 (55.8)	997 (59)	638 (67.4)	4653 (65.5)
	Joint	734 (33.1)	103 (12.4)	172 (23.1)	287 (42.1)	596 (35.3)	297 (31.4)	2189 (30.8)
	Extended	64 (2.9)	0 (0)	3 (0.4)	4 (0.6)	40 (2.4)	5 (0.5)	116 (1.6)
	Other	2 (0.1)	0 (0)	0 (0)	0 (0)	0 (0)	0 (0)	2 (0)

In addition, we observed that the prevalence of overweight, obesity, and hypertension among women showed an association with sociodemographic and other characteristics. We analyzed how overweight, obesity, and prehypertension varied when nonmodifiable factors such as age and modifiable factors such as educational level were considered in accordance with the revised consensus guidelines for India. A rising trend of obesity was observed with an increase in age. Women aged between 36 and 40 years (448/911, 49.2%) were more obese compared with those in other age groups. As expected, we observed the lowest percentage of obesity among women aged 18 to 23 years. The incidence of overweight was highest (341/1289, 26.45%) among women aged between 30 and 35 years and lowest among women aged between 18 and 23 years. In addition, we found a lower percentage of prehypertension (324/1946, 16.65%) among younger women aged between 18 and 23 years than in those aged between 30 and 35 years.

Using the revised consensus guidelines for India, we found varying proportions of BMI categories and prehypertension across rural and urban areas. Urban areas showed the highest proportions of women with obesity (775/1908, 40.62%), overweight (524/1908, 27.46%), and prehypertension (1008/1908, 52.83%) compared with women from rural areas. The proportion of underweight showed an almost decreasing trend with an increase in SES and vice versa for obesity rates. Upper-class and upper–middle-class women presented the lowest rates of underweight (17/308, 5.5% and 25/740, 3.4%, respectively). However, middle-class women presented a high incidence of underweight, obesity, and prehypertension (80/1331, 6.01%; 552/1331, 41.47%; and 738/1946, 37.92%, respectively). Women with a higher level of education (professional or honors) showed the lowest rates of overweight (6/34, 18%) and prehypertension (14/1946, 0.72%), and none were underweight. Women with a middle school education and illiterate women had higher obesity rates (299/665, 45% and 269/614, 43.8%, respectively). Women who had received a high school, graduate, or postgraduate education showed high rates of prehypertension (482/1946, 24.77% and 359/1946, 18.45%, respectively). Predictably, we observed high percentages of prehypertension (1409/1946, 72.4%) and overweight (706/2836, 24.89%) among unemployed women and the lowest rates of prehypertension (31/1946, 1.59%) among women who were either semiprofessional or professional (Table 3).

Table 4 shows the pattern of BMI and hypertension categories in each zone stratified by BMI, central obesity (WHtR and WC), and BP profiles. The comparative distribution of BMI categories of the 3877 women using the WHO and revised consensus guidelines for India are shown in Tables 4 and 5, in which regional variations are quite evident. Using WHO criteria and the revised consensus guidelines for India, the highest proportion of healthy women was observed in the Northeast (126/164, 76.8% and 99/164, 60.4%, respectively), and the proportion of overweight women was 42.1% (59/140) and 27.9% (39/140) in the East region, respectively. Accordingly, we found the highest rates of obesity in southern India (151/1072, 14.09% using the WHO criteria and 531/1072, 49.53% using the revised consensus BMI guidelines for India).

**Table 3:** Association between sociodemographic variables and BMI categories stratified by the revised consensus guidelines for India and stages of hypertension.

Variable		Underweight (n=204), n (%)	Healthy (n=1180), n (%)	Overweight (n=941), n (%)	Obese (n=1552), n (%)	Prehypertension (n=1946), n (%)
**Age group (years)**						
	18-23	72 (9.9)	314 (43.4)	140 (19.3)	198 (27.3)	324 (16.6)
	24-29	63 (6.6)	303 (31.8)	244 (25.6)	343 (36)	484 (24.9)
	30-35	45 (3.5)	340 (26.4)	341 (26.5)	563 (43.7)	673 (34.6)
	36-40	24 (2.6)	223 (24.5)	216 (23.7)	448 (49.2)	465 (23.9)
**Area of residence**						
	Rural	128 (6.5)	647 (32.9)	417 (21.2)	777 (39.5)	938 (48.2)
	Urban	76 (4)	533 (27.9)	524 (27.5)	775 (40.6)	1008 (52.8)
**Religion**						
	Hindu	109 (4.2)	747 (28.5)	677 (25.8)	1086 (41.5)	1323 (68)
	Muslim	73 (7.6)	308 (32)	199 (20.7)	383 (39.8)	525 (27)
	Christian	20 (9.7)	102 (49.3)	33 (15.9)	52 (25.1)	53 (2.7)
	Sikh	2 (2.4)	19 (22.9)	32 (38.6)	30 (36.1)	43 (2.2)
	Other	0 (0)	4 (80)	0 (0)	1 (20)	2 (0.1)
**Level of education**						
	Illiterate	38 (6.2)	161 (26.2)	146 (23.8)	269 (43.8)	379 (19.5)
	Primary school certificate	22 (6)	95 (25.8)	93 (25.3)	158 (42.9)	198 (10.2)
	Middle school certificate	31 (4.7)	180 (27.1)	155 (23.3)	299 (45)	321 (16.5)
	High school certificate	55 (5.8)	312 (32.8)	223 (23.4)	361 (38)	482 (24.8)
	Intermediate or post–high school diploma	32 (7.1)	155 (34.4)	104 (23.1)	160 (35.5)	193 (9.9)
	Graduate or postgraduate	26 (3.3)	263 (33.1)	214 (27)	291 (36.6)	359 (18.4)
	Profession or honors	0 (0)	14 (41.2)	6 (17.6)	14 (41.2)	14 (0.7)
**Socioeconomic status**						
	Upper class	17 (5.5)	87 (28.2)	79 (25.6)	125 (40.6)	133 (6.8)
	Upper-middle class	25 (3.4)	226 (30.5)	187 (25.3)	302 (40.8)	353 (18.1)
	Middle class	80 (6)	366 (27.5)	333 (25)	552 (41.5)	738 (37.9)
	Lower-middle class	48 (4.9)	331 (34)	222 (22.8)	372 (38.2)	461 (23.7)
	Lower class	34 (6.5)	170 (32.4)	120 (22.9)	201 (38.3)	261 (13.4)
**Occupation**						
	Unemployed	152 (5.4)	878 (31)	706 (24.9)	1100 (38.8)	1409 (72.4)
	Unskilled worker	19 (4.7)	102 (25.2)	98 (24.3)	185 (45.8)	218 (11.2)
	Semiskilled worker	19 (7.5)	84 (32.9)	53 (20.8)	99 (38.8)	137 (7)
	Skilled worker	8 (4.3)	57 (30.6)	37 (19.9)	84 (45.2)	93 (4.8)
	Arithmetic skill job	0 (0)	9 (33.3)	5 (18.5)	13 (48.1)	16 (0.8)
	Semiprofessional	4 (6.9)	19 (32.8)	13 (22.4)	22 (37.9)	31 (1.6)
	Professional	2 (2.2)	27 (30)	25 (27.8)	36 (40)	31 (1.6)

**Table 4:** Distribution of BMI stratified by the revised consensus guidelines for India and the World Health Organization (WHO) criteria along with stratified analysis of hypertension and various anthropometric indices of the study population.

Category and variable			North (n=2214), n (%)		Northeast (n=164), n (%)		East (n=140), n (%)		Central (n=126), n (%)		South (n=1072), n (%)		West (n=161), n (%)
**BMI category (revised Indian guidelines)**													
	Underweight (<18.5 kg/m^2^)	96 (4.3)		13 (7.9)		3 (2.1)		15 (11.9)		56 (5.2)		21 (13)	
	Healthy (18.5-22.9 kg/m^2^)	695 (31.4)		99 (60.4)		36 (25.7)		55 (43.7)		240 (22.4)		55 (34.2)	
	Overweight (23-24.9 kg/m^2^)	582 (26.3)		27 (16.5)		39 (27.9)		19 (15.1)		245 (22.9)		29 (18)	
	Obese (≥25 kg/m^2^)	841 (38)		25 (15.2)		62 (44.3)		37 (29.4)		531 (49.5)		56 (34.8)	
**BMI category (WHO criteria)**													
	Underweight (<18.5 kg/m^2)^	96 (4.3)		13 (7.9)		3 (2.1)		15 (11.9)		56 (5.2)		21 (13)	
	Healthy (18.5-24.9 kg/m^2^)	1277 (57.7)		126 (76.8)		75 (53.6)		74 (58.7)		485 (45.2)		84 (52.2)	
	Overweight (25-29.9 kg/m^2^)	699 (31.6)		25 (15.2)		59 (42.1)		33 (26.2)		380 (35.4)		44 (27.3)	
	Obese (≥30 kg/m^2^)	142 (6.4)		0 (0)^a^		3 (2.1)		4 (3.2)		151 (14.1)		12 (7.5)	
**Waist category**													
	Central_obesity_WHtr^b^	1192 (53.8)		86 (52.4)		27 (19.3)		80 (63.5)		481 (44.9)		97 (60.2)	
	Central_obesity_WC^c^	498 (22.5)		13 (7.9)		7 (5)		38 (30.2)		164 (15.3)		48 (29.8)	
**Hypertension category (JNC 8^d^ criteria)**													
	Normotensive	1173 (53)		134 (81.7)		70 (50)		73 (57.9)		363 (33.9)		118 (73.3)	
	Prehypertension	1041 (47)		30 (18.3)		70 (50)		53 (42.1)		709 (66.1)		43 (26.7)	

^a^No participant identified in this category.

^b^WHtR: waist-to-height ratio.

^c^WC: waist circumference.

^d^JNC 8: Eighth Joint National Committee on Prevention, Detection, Evaluation, and Treatment of High Blood Pressure.

**Table 5:** Comparative distribution of BMI categories (obese, overweight, healthy, and underweight) of the women using the World Health Organization (WHO) criteria and the revised consensus guidelines for India (n=3877).

			Participants, n (%)
**BMI (WHO)**			
	Obese	312 (8.05)	
	Overweight	1240 (31.98)	
	Healthy	2121 (54.71)	
	Underweight	204 (5.26)	
**BMI (Indian)**			
	Obese	1552 (40.03)	
	Overweight	941 (24.27)	
	Healthy	1180 (30.44)	
	Underweight	204 (5.26)	

A clear variation in the frequency of central obesity, WHtR, and WC was noted among women across the regions. The central obesity WHtR and WC were highest among Central Indian women (80/126, 63.5% and 38/126, 30.2%, respectively). We also analyzed how BP profile varied across regions using JNC 8 criteria. After stratifying by region, our analysis showed that the women who were enrolled as healthy controls in the study either were normotensive (healthy BP profile) or fell under the prehypertension category. The Northeast region (Shillong) showed the highest incidence of normotensive women (134/164, 81.7%) and the lowest rates of women with prehypertension (30/164, 18.3%). However, prehypertension was found to be highest among South Indian women (709/1072, 66.14%), followed by women from East (70/140, 50%), North (1041/2214, 47.02%), and Central (53/126, 42.1%) India, as well as 26.7% (43/161) from the Western region of the country. Table 6 shows the multivariate logistic regression analysis on the association between demographic characteristics (age, region, residence, occupation, SES, education, marital status, and religion of participants) and general and central obesity. We observed that age, region, religion, and occupation were significantly associated with obesity. Obesity was significantly higher in older age groups (36-40 y; odds ratio [OR] 2.57, 95% CI 1.906-3.471; *P*<.001), central regions (OR 1.76, 95% CI 1.144-2.719; *P*=.01), and the Muslim population (OR 2.73, 95% CI 2.245-3.313; *P*<.001).

**Table 6:** Multivariate logistic regression analysis on the association between demographic characteristics (age, region, residence, occupation, socioeconomic status, education, marital status, and religion of the participants) and general and central obesity.

Characteristic		Central obesity (based on WC^a^)			General obesity (based on WHO^b^ guidelines)	
		OR^c^ (95% CI)	*P* value	OR (95% CI)		*P* value
**Age group (years)**						
	18-23^d^	N/A^e^	N/A	N/A		N/A
	24-29	1.199 (0.909-1.582)	.20	1.082 (0.725-1.1615)		.70
	30-35	1.574 (1.184-2.091)	.002	1.132 (0.755)-1.699)		.55
	36-40	2.572 (1.906-3.471)	<.001	1.334 (0.871-2.044)		.19
**Region**						
	North^d^	N/A	N/A	N/A		N/A
	Northeast	0.462 (0.196-1.086)	.08	0 (0-0)		>.99
	East	0.180 (0.081-0.400)	<.001	0.274 (0.083-0.899)		.03
	Central	1.764 (1.144-2.719)	.01	0.406 (0.144-1.146)		.09
	South	0.686 (0.549-0.858)	.001	2.116 (1.587-2.823)		<.001
	West	1.284 (0.877-1.879)	.20	0.861 (0.453-1.635)		.65
**Residence**						
	Rural^d^	N/A	N/A	N/A		N/A
	Urban	0.757 (0.643-0.890)	.001	0.642 (0.507-0.811)		<.001
**Occupation**						
	Unemployed^d^	N/A	N/A	N/A		N/A
	Unskilled worker	0.829 (0.597-1.149)	.26	1.240 (0.840-1.831)		.28
	Semiskilled worker	1.101 (0.787-1.540)	.57	0.787 (0.454-1.364)		.39
	Skilled worker	1.088 (0.757-1.564)	.65	0.968 (0.547-1.713)		.91
	Arithmetic skill job	0.974 (0.370-2.563)	.96	2.058 (0.673-6.288)		.21
	Semiprofessional	1.718 (0.938-3.145)	.08	1.424 (0.606-3.348)		.42
	Professional	1.028 (0.597-1.771)	.92	2.275 (1.103-4.695)		.03
	Other	4.029 (1.614-10.062)	.003	2.168 (0.484-9.716)		.31
**Socioeconomic status**						
	Upper class^d^	N/A	N/A	N/A		N/A
	Upper-middle class	0.518 (0.388-0.691)	<.001	0.384 (0.252-0.584)		<.001
	Middle class	0.388 (0.295-0.509)	<.001	0.352 (0.242-0.512)		<.001
	Lower-middle class	0.431 (0.326-0.568)	<.001	0.343 (0.234-0.504)		<.001
	Lower class	0.477 (0.352-0.647)	<.001	0.266 (0.167-0.424)		<.001
**Educational level**						
	Illiterate^d^	N/A	N/A	N/A		N/A
	Primary school certificate	0.815 (0.582-1.142)	.24	0.666 (0.425-1.043)		.08
	Middle school certificate	1.083 (0.828-1.417)	.56	0.722 (0.504-1.036)		.08
	High school certificate	1.076 (0.843-1.372)	.56	0.697 (0.501-0.968)		.03
	Intermediate or post–high school diploma	0.823 (0.596-1.137)	.24	0.555 (0.352-0.875)		.01
	Graduate or postgraduate	1.161 (0.885-1.521)	.28	0.497 (0.333-0.742)		.001
	Profession or honors	1.714 (0.758-3.873)	.20	0.473 (0.145-1.544)		.22
**Marital status**						
	Unmarried^d^	N/A	N/A	N/A		N/A
	Married	1.136 (0.887-1.455)	.31	1.136 (0.783-1.648)		.50
	Un-remarried	1.081 (0.441-2.654)	.86	0.167 (0.019-1.485)		.11
	Widowed	1.085 (0.442-2.664)	.86	0.625 (0.174-2.241)		.47
**Religion**						
	Hindu^d^	N/A	N/A	N/A		N/A
	Muslim	2.728 (2.245-3.313)	<.001	1.133 (0.844-1.521)		.41
	Christian	1.027 (0.534-1.974)	.94	0.312 (0.094-1.037)		.06
	Sikh	0.897 (0.515-1.563)	.70	0.806 (0.313-2.074)		.65
	Other	1.687 (0.145-19.598)	.68	0.000 (0.000-)		>.99

^a^WC: waist circumference.

^b^WHO: World Health Organization.

^c^OR: odds ratio.

^d^Reference group.

^e^N/A: not applicable.

### Correlation Between BMI and Overweight Indicators

**Figure 2:**
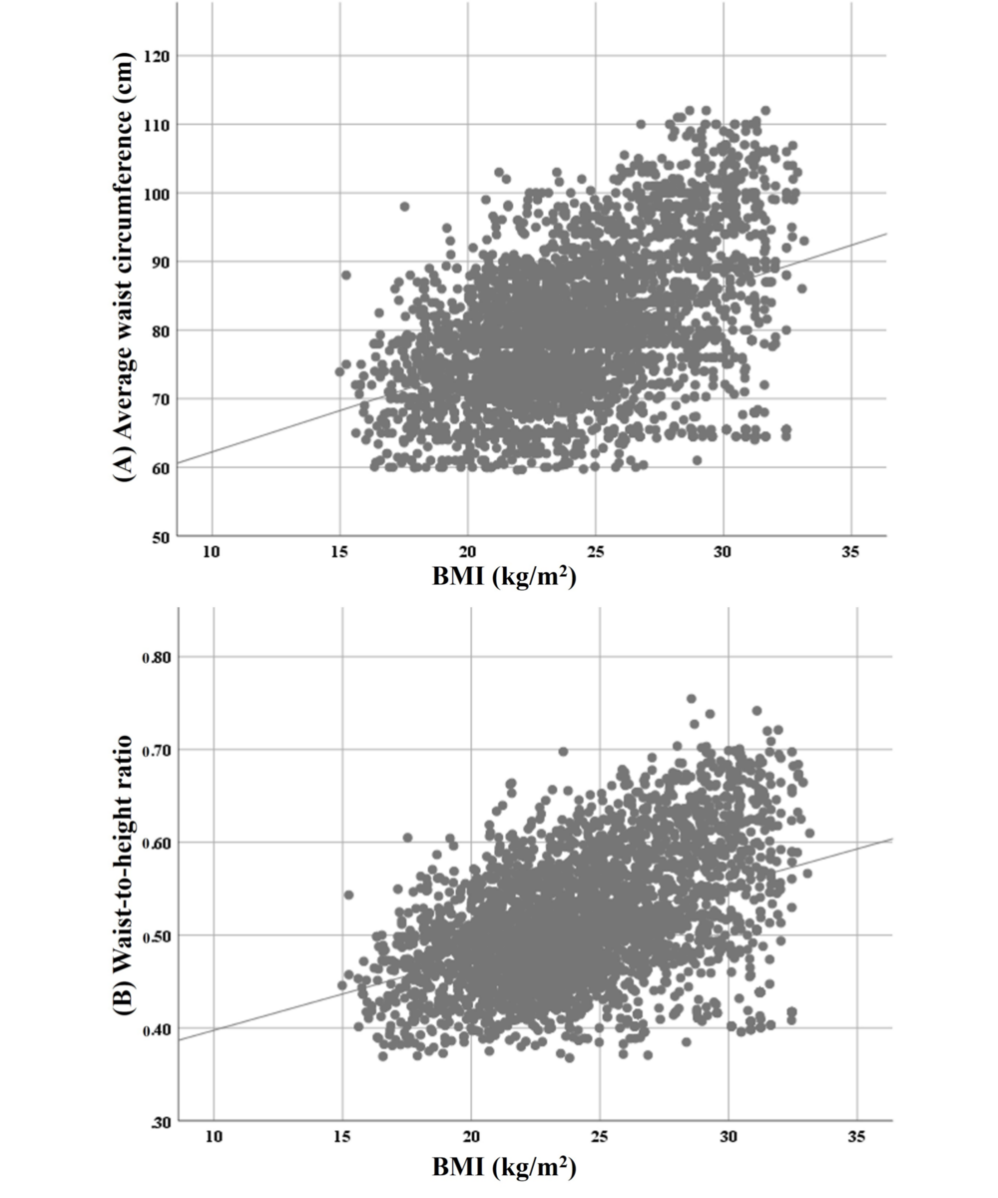
Correlation between BMI and (A) waist circumference and (B) waist-to-height ratio.

Pearson correlation analysis was conducted, and we observed a positive but weak association between these variables. Figure 2 illustrates the correlation between BMI and (1) WC and (2) WHtR of the participants.

#### BMI and WC

BMI and WC showed a statistically significant linear relationship (*r*=0.417; *P*<.001). The direction of the relationship was positive, meaning that these variables tended to increase together (ie, greater WC was associated with greater BMI). However, the magnitude, or strength, of the association was relatively weak (0.3<|*r*|<0.5).

#### BMI and WHtR

Similarly, BMI and WHtR showed a statistically significant linear relationship (*r*=0.422; *P*<.001). The direction of the relationship was positive, implying that these variables tended to increase together (ie, greater WHtR was associated with greater BMI). However, the magnitude, or strength, of the association was relatively weak (0.3<|*r*|<0.5).

## Discussion

### Principal Findings

The ongoing ICMR-PCOS Task Force study is the first pan-Indian study conducted among urban and rural women across representative geographical regions. To the best of our knowledge, this is the first report on the pattern and prevalence of overweight, obesity, and hypertension among apparently healthy women with region-wise categorization into North, East, West, South, Northeast, and Central India. This part of the study uses a specific scenario of the risks to women’s health, such as overweight and obesity, influenced by multiple determinants of health. Although the prevalence of overweight and obesity affects all segments of the population, there are notable trends among certain sections wherein women are more likely to be obese or gain weight. There are a number of studies available in the literature that use the WHO criteria for BMI cutoffs for defining overweight and obesity prevalence in India. However, these are scientifically challenged as these guidelines are based on studies of White individuals (defined as BMI ≥30 for obesity). Our study provides the comparative estimates of overweight and obesity based on the revised consensus guidelines for India, that is, it defines BMI as underweight (<18.5 kg/m^2^), healthy (18.5-22.9 kg/m^2^), overweight (23.0-24.9 kg/m^2^), and obese (≥25 kg/m^2^).

Our results showed that overweight, obesity, and hypertension are also prevalent among apparently healthy women across the country. Health concerns in women start to creep in when entering their second and third decades of life. Our results also showed a rising trend of obesity with an increase in age. While going through age categories, we found that women aged between 36 and 40 years were more obese (448/911, 49.2%). Overweight was observed more among women aged between 30 and 35 years. A significant proportion of women from other age groups was found to be either overweight or obese. The rising incidence of overweight and obesity is a challenge across all age groups [24].

Our findings showed a high proportion of healthy women across rural and urban areas using the revised Indian criteria (647/1969, 32.86% and 533/1908, 27.94%, respectively). These findings are in agreement with the statement under “Patterns by background characteristics of NFHS-5,” which states that the proportion of healthy women was higher in rural areas than in urban areas. Our results show higher rates of obesity among urban women than in women from rural areas. These results are in agreement with the data analysis of the NFHS for the epidemiology of obesity in Indian adults and similar to the findings of other studies [9,25-28]. Mishra et al [29] also revealed that the urban population was at a higher risk of obesity than the rural population, in consonance with our findings. We believe that lifestyle and behavioral factors are the main reasons for the differences between urban and rural populations.

Our results showed that both obesity and overweight are significantly prevalent, along with cross-regional variations. The revised consensus guidelines for BMI in Indians showed an increased proportion of obese women in South India (531/1072, 49.53%), followed by East (62/140, 44.3%) and North (841/2214, 37.99%) India, whereas Northeast India showed the lowest rates of obesity (25/164, 15.2%). These findings are partially in agreement with the findings of the NFHS-5, which reported the highest proportion of obese women in Puducherry (south), followed by Chandigarh and Delhi (north). These cross-regional variations highlight the role of geographical factors in these disorders. Levels of urbanization may be the reason for the lower prevalence in the northeastern and eastern regions than in other regions. The northeastern, eastern, and central regions are the 3 least urbanized regions in India [30]. A systematic review reported that variations in the prevalence of obesity in India are due to diverse geographical conditions, lifestyles, and dietary patterns. As expected, we found that only a small proportion of women belonging to the upper class were underweight, and a good proportion (125/308, 40.6%) of women from the same class presented obesity. These findings are similar to those of a cross-sectional study that reported high rates of overweight and obesity (71.8%) among the socioeconomically well-off community of Marwaris, West Bengal, India. This can be attributed to their sedentary lifestyle and high-calorie food intake compared with women of lower SES. Obesity is a triggering factor for various diseases; we used Homeostatic Model Assessment of IR as an indicator of IR and found that 49.77% (661/1328) of participants had IR, with Homeostatic Model Assessment of IR of >2.

Our results showed that women with the highest level of education (with a profession or honors) were not underweight and showed the lowest rates of overweight (6/34, 18%) and prehypertension (14/1946, 0.72%) incidence compared with women who were illiterate or had other levels of education. These trends resemble the findings in other regions of the world, wherein a better education appears to be associated with a lower likelihood of obesity, especially among women. Data analyses of individual-level national health surveys in countries such as Australia, Canada, England, and Korea also reported a linear relationship between the number of years spent in full-time education and the probability of obesity, with most educated individuals displaying lower rates of the condition [31]. Several other studies have reported a strong education gradient in BMI or obesity, with better educated women being less likely to be overweight or obese [32-34]. Our results suggest that general education campaigns and the design and implementation of effective policies can play a significant role in reducing obesity to curb the epidemic.

Hypertension is of particular importance to public health because of its association with a number of diseases contributing to premature deaths. The incidence of hypertension has increased drastically in many LMICs, including India. Our analysis showed patterns of higher incidence of prehypertension among urban women compared with rural women, and a rising trend was observed with an increase in age. These findings refute the age-old concept that hypertension is not a major problem in rural India. The increasing trend of prehypertension with age is in agreement with the NFHS-5, which reported an increase in all categories of hypertension, including prehypertension, with age for both women and men. We also found patterns of greater prevalence of prehypertension among women belonging to the middle class (738/1946, 37.92%) and the lower middle class (461/1946, 23.69%) than among women from the upper class, with an incidence rate of 6.89% (133/1931). This may be linked to the tendencies toward sedentary living and eating unhealthy foods, among other things. These findings are in contradiction to the findings of studies from Nigeria and Kenya that reported a greater prevalence of hypertension among wealthier groups [35,36]. Prehypertension was found to be more prevalent in women who had a lower educational level (high school) and women who were illiterate (379/1946, 19.48%) compared with women with higher levels of education (professional or honors). These findings support the belief that a higher educational level increases awareness and information regarding hypertension and, subsequently, encourages adaptation to healthy lifestyles, and that could be the possible reason behind it. Reddy et al [37] reported based on a cross-sectional study that hypertension was significantly more prevalent in the group with a lower educational level compared with that with a higher educational level. Our results showed that age, level of education, occupation, geographical background, and SES were significant predictors of hypertension. The possible explanations for the lower incidence rates of overweight, obesity, and hypertension in women with a higher education and from the upper socioeconomic class in this study may be attributed to good awareness of preventive measures, better adherence to medical advice, and lifestyle modifications. We believe that there is an urgent need to implement strategies to increase awareness of hypertension and promote healthier lifestyles.

Region-wise categorization into North, East, West, South, Central, and Northeast India allowed us to capture and present the regional estimates of prehypertension and BMI categories among healthy subsets of women, which showed the highest rates of prehypertension among women from southern parts of India (709/1072, 66.14%) followed by East Indian women (70/140, 50%) and the lowest rates among Northeast Indian women. Overall, the identification of women who might face greater challenges with their disease management suggests a role for relevant health authorities in executing appropriately targeted public health measures and policies to ensure good hypertension management in this population.

### Strengths

The major strength of our study is its large sample size with apparently healthy reproductive-age women recruited from the 6 regions of India. Hence, it provides region-specific incidences of overweight, obesity, and prehypertension. The findings of our study may be used to plan interventions or policies for the prevention and control of obesity and hypertension among different populations. The results of this study should be interpreted in the context of the potential limitations. Furthermore, we adopted a common instruction manual and standard equipment of similar specifications at all study centers to ensure the accuracy and reliable quality of all the measurements throughout the entire study period so as to generate quality data.

### Limitations

There are some limitations to our study. First, to overcome population heterogeneity, we attempted to divide the study areas geographically into North, East, West, South, Northeast, and Central India and arrive at region-specific rates. However, the cultural and dietary practices vary drastically state-wise, and this may have a bearing on the results. Second, we did not report on the awareness and control of obesity and BP and the association of sociodemographic factors such as SES and nutrition with height. We used the BG Prasad social classification, which is applicable to rural and urban areas, but it only considers income and excludes the other SES criteria. In addition, this study provides estimates from 6 regions that include only a few states and union territories and did not include several major Indian states such as Rajasthan and Madhya Pradesh. Above all, the findings are limited to only feminine (female individuals) aged between 18 and 40 years.

### Conclusions

To conclude, overweight, obesity, and hypertension epidemics are highly prevalent among healthy subsets of reproductive-age Indian women across rural and urban strata with cross-regional variations. Age, level of education, geographical background, and SES were found to be predictors of BMI and hypertension categories. Genetic and environmental factors such as diet and activity level are likely contributors to these aberrations; however, role of the type of family and marital status need to be explored further. Our results highlight the importance of both nonmodifiable (such as age) and modifiable (such as educational level) factors in determining patterns of overweight, obesity, and hypertension. Education plays an important role in tackling overweight and obesity. This study may serve as an indicator providing early clues and recommending further research using more rigorous scientific methods. In light of our findings, there is a need for targeted attention to both prevent future epidemics in a population that has not developed obesity or hypertension and help maintain healthy weight and BP in individuals with hypertension. Public health awareness regarding overweight, obesity, and BP needs to be tailored differently for women considering the regional, rural, and urban dimensions. We advocate for multidisciplinary efforts to address these issues, and policy makers need to consider what level of evidence should be deemed sufficient to prompt action and tackle these issues.

## Abbreviations

BP: blood pressure
DBP: diastolic blood pressure
ICMR: Indian Council of Medical Research
IR: insulin resistance
JNC 8: Eighth Joint National Committee on Prevention, Detection, Evaluation, and Treatment of High Blood Pressure
LMICs: low- and middle-income countries
NCD: noncommunicable disease
NFHS: National Family Health Survey
OR: odds ratio
PCOS: polycystic ovary syndrome
SBP: systolic blood pressure
SES: socioeconomic status
WC: waist circumference
WHO: World Health Organization
WHR: waist-to-hip ratio
WHtR: waist-to-height ratio

## Data Availability

The data used and analyzed during this study are available from the chief coordinator of the study and can be obtained upon reasonable request.
